# Warm Weather Dietetics: The Scientific Apportioning of a Summer Diet

**Published:** 1913-06-28

**Authors:** 


### WARM WEATHER DIETETICS.

### The Scientific Apportioning of a Summer Diet.

When there is a change of seasons the lay press is always active in foisting upon its readers the views of amateur dietitians with regard to what should be eaten and what eschewed if the individual wishes to be comfortably in harmony with his climatic environment. To the expert such lay disquisitions are often amusing, but they have their serious side as well, for undoubtedly a great deal of this well-meant advice is directly prejudicial to the health of the unlucky man who binds his faith to what the lay dietitian asserts. As an example of such advice we may take the sample menu that has lately been drawn up by a well-known London daily as an ideal bill of fare for a luncheon on a hot summer's day. This included, if we remember rightly, such dishes as salmon mayonnaise and strawberries and cream; its total caloric value, worked out on the basis of a small quantity of each dish, amounts to more than double that which the average adult requires for a full day's nourishment. It is, therefore, a brilliant example of *luxus* consumption in its worst form; but simply because it has the authority of a "well-known *chef*" and of the daily paper behind it, there are doubtless many individuals who have ordered it under the impression that they are ordering the best and most wholesome summer fare obtainable.

As a matter of fact the scientific apportioning of food in summer is regulated by exactly the same rules as those that hold good in winter time. The basis of such apportioning is to strike a happy mean between the demands of the body and the desire of the appetite. In winter, when body metabolism is more active in order to give the organism more heat, a larger quantity of heat-producing food must be consumed in order to make good the tissue deficiency. In summer, tissue metabolism is less active and, consequently, less heat-producing food is required; possibly the amount of tissue-forming food, as distinct from heat-producing food, is the same both for summer and winter requirements, provided the individual does the same work in both seasons. This simple rule will enable the layman to draw up his own bill of fare and to vary it according to his needs, so long as he has before him the caloric coefficients of the various articles of diet ordinarily consumed. These are now given in many popular manuals, and it is relatively easy to look them up. In general it may be stated that the carbohydrates and the fats are the main heat-producing foodstuffs, while the proteids are the tissue formers which cannot be avoided without detriment to the body.

In summer, therefore, the meals should be smaller, less rich in sugars and starches and fats than in winter. It is easy to see that this simple rule is almost invariably disregarded in popular disquisitions on the summer diet. Certain conventions have become so firmly established that no amount of scientific criticism will shake them in popular estimation. Salmon mayonnaise, perhaps the worst possible dish for summer consumption, is as firmly rooted in popularity as is the summer's morning meal at which figure such heat-producing dishes as bacon and eggs, buttered toast, and porridge. Yet even with regard to breakfast it is gratifying to note that the influence of our American cousins is making itself felt. A breakfast of cereal and fruit, with black coffee, a cup of China tea—since it is hopeless to expect that we will have the courage to revert to the custom of our ancestors and allow wine or light beer to figure on the breakfast table—or water cocoa, is an ideal commencing meal in summer. On this the average city man or sedentary worker will easily support life and cheerful spirits until luncheon time. Luncheon itself may be anticipated with advantage by a cup of tea and a biscuit or some light refreshment partaken at eleven o'clock. During the heat of the day there is a not unnatural desire to consume large quantities of water, preferably iced. For those whose stomachs can stand this treatment, the custom has its advantages, but it is well to point out that there is considerable scientific warrant for what may be called the homœopathic system of drinking in summer. This system is well recognised in tropical countries where hot drinks are partaken of during the warm weather. Tea with a slice of lemon, or better still ginger, in it is both an admirable thirst-quencher and body cooler. A light, acid wine, taken at body temperature, is equally efficient in both respects, and far more so than a glass of beer or a cup of coffee. Milk should be eschewed, unless the consumer is absolutely certain that it is fresh and above suspicion; in hot weather a glass of London milk is usually a swarming colony of pathological organisms. Luncheon itself may be made up of three light courses, with fruit to follow. Thin soup, hot or cold as preferred, is a good commencing dish; the second course may be fish or entrée, and the third joint or game, with a salad or with such vegetables as are in season. Asparagus, for example, is one of the best vegetables that can be taken in summer; the amount of nourishment it contains is small, but it is satisfying without being unduly heat-producing, especially when eaten cold with an acid sauce. Squashes and vegetable marrows are similarly excellent and can be served in various ways. A vegetable luncheon is admirable in summer, provided that the consumer eschews all those makeshifts to which our vegetarian restaurants are so prone in their desire to give their courses a more or less—usually less—non-vegetarian appearance.

Dinner will, of course, be taken in the evening whenever possible. A four-course meal here is perfectly allowable, for with care the menu can be so framed that the caloric value of the viands is not above that required, while at the same time the demands of a healthy appetite are worthily met. Soup, again, is the best for the first course; it should, of course, be a consommé, unthickened, and carefully skimmed. Fish in season and an entrée should follow. A common mistake is to enrich the sauces for both with heat-producing materials, of which cheese is the favourite; the Italian method, in which tomato sauce takes the place of cheese, is far to be preferred. A good joint—for all that the renowned *chef* may say against it—remains the best middle course. With this should invariably be served the salad with its simple oil and vinegar dressing; to enbalm the salad vegetables, whether cooked or uncooked, in a rich sauce of egg and cream is to spoil the whole menu framed with a due regard to the health and comfort of the diner. The sweets at the end should show a minimum of starch and sugar.

